# TDP-43 Inclusion Bodies Formed in Bacteria Are Structurally Amorphous, Non-Amyloid and Inherently Toxic to Neuroblastoma Cells

**DOI:** 10.1371/journal.pone.0086720

**Published:** 2014-01-30

**Authors:** Claudia Capitini, Simona Conti, Michele Perni, Francesca Guidi, Roberta Cascella, Angela De Poli, Amanda Penco, Annalisa Relini, Cristina Cecchi, Fabrizio Chiti

**Affiliations:** 1 Section of Biochemistry, Department of Biomedical, Experimental and Clinical Sciences, University of Florence, Florence, Italy; 2 Department of Physics, University of Genoa, Genoa, Italy; International Centre for Genetic Engineering and Biotechnology, Italy

## Abstract

Accumulation of ubiquitin-positive, tau- and α-synuclein-negative intracellular inclusions of TDP-43 in the central nervous system represents the major hallmark correlated to amyotrophic lateral sclerosis and frontotemporal lobar degeneration with ubiquitin-positive inclusions. Such inclusions have variably been described as amorphous aggregates or more structured deposits having an amyloid structure. Following the observations that bacterial inclusion bodies generally consist of amyloid aggregates, we have overexpressed full-length TDP-43 and C-terminal TDP-43 in *E. coli*, purified the resulting full-length and C-terminal TDP-43 containing inclusion bodies (FL and Ct TDP-43 IBs) and subjected them to biophysical analyses to assess their structure/morphology. We show that both FL and Ct TDP-43 aggregates contained in the bacterial IBs do not bind amyloid dyes such as thioflavin T and Congo red, possess a disordered secondary structure, as inferred using circular dichroism and infrared spectroscopies, and are susceptible to proteinase K digestion, thus possessing none of the hallmarks for amyloid. Moreover, atomic force microscopy revealed an irregular structure for both types of TDP-43 IBs and confirmed the absence of amyloid-like species after proteinase K treatment. Cell biology experiments showed that FL TDP-43 IBs were able to impair the viability of cultured neuroblastoma cells when added to their extracellular medium and, more markedly, when transfected into their cytosol, where they are at least in part ubiquitinated and phosphorylated. These data reveal an inherently high propensity of TDP-43 to form amorphous aggregates, which possess, however, an inherently high ability to cause cell dysfunction. This indicates that a gain of toxic function caused by TDP-43 deposits is effective in TDP-43 pathologies, in addition to possible loss of function mechanisms originating from the cellular mistrafficking of the protein.

## Introduction

TAR DNA-binding protein 43 (TDP-43) is a multifunctional nuclear protein initially described as a transcription factor [1], but later found to be also involved in regulation of RNA splicing, microRNA processing, mRNA transport, stability and translation [2], [3]. In 2006 it was reported, for the first time, that TDP-43 is the main component of the ubiquitin-positive, tau-negative and α-synuclein-negative protein inclusions accumulating in the fronto-temporal cortex and hippocampus of the brain and in the motor neurons of the spinal cord of patients suffering from frontotemporal lobar degeneration with ubiquitin-positive inclusions (FTLD-U) and amyotrophic lateral sclerosis (ALS) [4]. TDP-43 inclusions are also found in other neurodegenerative conditions, such as Alzheimer’s disease and Parkinson’s disease, as well as in inclusion body myositis and myofibrillar myopathy [5]. However, the presence of other protein inclusions and clinical manifestations in all such conditions, and the observation that all TDP-43 mutations so far discovered are only associated with familial ALS and FTLD-U, suggest that TDP-43 aggregation is rather a secondary process in this group of neurodegenerative and muscle diseases [5].

Pathological TDP-43 aggregation is associated with a dislocation of this protein from the nucleus, where the protein normally resides and plays its functions, to the cytoplasm, where the inclusions accumulate [4], [6], [7]. In such cytoplasmic inclusions TDP-43 is hyperphosphorylated, ubiquitinated and cleaved to form C-terminal fragments [4], [6], [7], although in the spinal cord motor neurons the inclusions consist rather of full-length TDP-43 [8].

The structure of TDP-43 in the inclusions of ALS and FTLD-U patients is not yet clear and subject of current debate. In particular, it is not yet clear whether TDP-43 inclusions consist of amyloid fibrils or rather another type of protein aggregate. In order to be classified as amyloid, protein aggregates need to comply with three main criteria that are nowadays accepted by investigators from different disciplines, from biophysicists to clinicians: the presence of a fibrillar morphology with the fibrils having a diameter of typically 7–13 nm, the presence of a cross-β secondary structure and the binding to amyloid-diagnostic dyes like Congo red (CR), thioflavin T (ThT) and thioflavin S (ThS). Spinal cord sections of ALS patients show the presence of TDP-43 positive, 10–20 nm wide filaments in the absence of CR and ThS binding, thus suggesting a non-amyloid structure [9]–[11]. However, a very recent report indicates the presence of a widespread remarkable ThS staining in TDP-43 inclusions present in the lower motor neurons of sporadic ALS cases, suggesting rather an amyloid-like structure [12]. In another very recent report, it was shown that a few TDP-43 inclusions of ALS patients may consist of 10–20 nm fibrils able to bind ThS, but such features were found only in a small fraction of skein-like inclusions of the spinal cord, with amyloid-like characteristics being absent in most spinal cord skeins and absent altogether in other TDP-43 inclusions of the spinal cord and in all inclusions of the brain [13].

As far as FTLD-U is concerned, TDP-43 inclusions in the brain were found to consist of 15–20 nm wide filaments [14]. In another report, the dentate gyrus of FTLD-U patients was shown to contain 10–17 nm filaments in intranuclear, cytoplasmic and neuritic inclusions, with the latter containing filaments that were randomly oriented [10]. Similarly, filaments with a mean diameter of 9 nm and 10 nm were found in cytoplasmic and neuritic inclusions, respectively, of FTLD-U brains, whereas fibrils with a mean diameter of 18 nm were present in intranuclear inclusions [15]. Inclusions present in the brain of these patients showed an inability to bind amyloid-specific dyes [16], [17], a finding confirmed in the spinal cords of FTLD-U patients, where no ThS positive inclusions were found [13]. However, in the same study showing a large ThS positivity in ALS spinal cords, a remarkable and diffuse ThS staining of TDP-43 inclusions in FTLD-U brains was also reported [12].

Likewise, studies *in vitro* describing aggregation of purified full-length or fragmented TDP-43 have reported conflicting reports as to the structure of the resulting TDP-43 aggregates. In a first report, full-length TDP-43 was shown to form filaments unable to bind CR and ThT [18]. By contrast, other reports have shown that peptides encompassing the most highly aggregating region of C-terminal TDP-43 are able to form ∼ 10 nm fibrils with β-sheet secondary structure and dye binding [19]–[22].

In this work we have addressed this point by overexpressing full-length and C-terminal TDP-43 in *E. coli*, purifying the resulting TDP-43 containing inclusion bodies (FL TDP-43 IBs and Ct TDP-43 IBs, respectively) and subjecting them to a number of biophysical analyses to assess their structure and morphology. Indeed, it is increasingly accepted that bacterial IBs mainly consist of amyloid-like aggregates [23]–[28]. Therefore, the detection of amyloid structure in the TDP-43 aggregates present in FL TDP-43 IBs and Ct TDP-43 IBs could be informative on the intrinsic propensity of this protein to form this type of protein aggregate. By contrast, the absence of amyloid-like structure in bacterial IBs would suggest a propensity to form another type of deposit. In addition to performing a biophysical investigation of the TDP-43 IBs, we have transfected eukaryotic cell cultures with FL TDP-43 IBs to evaluate their inherent toxicity. We will show that the TDP-43 aggregates contained in the bacterial IBs do not bind ThT and CR, possess random coil and β-turn secondary structure, and are highly susceptible to proteinase K (PK) digestion, thus possessing none of the amyloid distinctive hallmarks. In such unstructured/amorphous form, however, the FL TDP-43 aggregates appear to be highly toxic when transfected to the cytosol of the cultured cells, revealing their inherent ability to cause cell dysfunction.

## Materials and Methods

### Materials

All reagents were of analytical grade or of the highest purity available. Amyloid peptide 1–42 (Aβ_42_), Dulbecco’s modified eagle medium (DMEM), fetal bovine serum (FBS), hen egg white lysozyme (HEWL), pluronic acid F-127, ampicillin, ThT, CR, PK, carbobenzoxy-Leu-Leu-leucinal (MG-132), 3-methyladenine (3-MA), chloroquin and other chemicals were from Sigma-Aldrich, unless otherwise stated. 2′,7′-dichlorodihydrofluorescein diacetate (CM-H_2_DCFDA; Molecular Probes, Milan, Italy) was prepared as stock solutions in dimethylsulfoxide (DMSO), dried under nitrogen and stored in light-protected vessels at −20°C until use. Aβ_42_ oligomers were prepared as previously described [29] and resuspended in F-12 HAM to different concentrations ranging from 7.5 µg/mL to 215 µg/mL.

### FL and Ct TDP-43 Gene Cloning and Protein Expression

The genes for FL TDP-43 (residues 1–414) and its C-terminal fragment (residues 208–414) were cloned downstream of the glutathione S-transferase (GST) gene in the pGEX-2T plasmid. In brief, the sequence coding for FL TDP-43 and that coding for Ct TDP-43 were amplified from the pINCY vector (Thermo Fisher Scientific, Waltham, MA, USA) by PCR, using forward and reverse primers containing the restriction sites for *BamHI* and *EcoRI* enzymes, respectively. Each amplified sequence and the pGEX-2T plasmid were digested with *BamHI* and *EcoRI* (Thermo Fisher Scientific) and combined with the T4 DNA ligase (Thermo Fisher Scientific) to obtain the constructs coding for the GST/FL TDP-43 and the GST/Ct TDP-43 fusion proteins. Their correct nucleotide sequence was verified by DNA sequencing.

Cultures of *E. coli* XL1 Blue cells (Agilent Technologies, Santa Clara, CA, USA) were transformed with the resulting plasmids containing FL TDP-43 or Ct TDP-43 and were grown overnight at 37°C in LB medium with 100 µg/mL ampicillin under vigorous shaking. Cells were then diluted 1∶10 in fresh medium and grown at 37°C until OD_600 nm_ ∼ 0.6. Protein expression was induced using 1 mM isopropyl β-D-1-thiogalactoside (IPTG; Inalco, Paris, France). Cells were harvested by centrifugation, resuspended in PBS buffer (137 mM NaCl, 2.7 mM KCl, 4.3 mM Na_2_HPO_4_, 1.4 mM KH_2_PO_4_, 1 mM EDTA, 1 mM β-mercaptoethanol, 0.1 mM PMSF, at pH 7.3) and then lysed by 30 min incubation with 1 mg/mL HEWL in ice, followed by sonication at 40 kHz (five cycles of 30 s each). The expression of FL and Ct TDP-43 and their presence in the supernatant or in the *pellet* fractions after cell lysis were checked by SDS-PAGE, using 12% (w/v) polyacrylamide gels. Wt AcPDro2 IBs and C43S AcPDro2 IBs were purified and analysed as described in Methods S1.

### IBs Purification

IBs were purified from IPTG induced cells harbouring the pGEX-2T/FL TDP-43 plasmid, the pGEX-2T/Ct TDP-43 plasmid and the only pGEX-2T plasmid by detergent-based procedures. Briefly, cells obtained from 1 L cultures were harvested by centrifugation at 29000×g for 15 min at 4°C, resuspended in 40 mL of lysis buffer (50 mM Tris-HCl, 100 mM NaCl, 1 mM EDTA, at pH 8.0) and maintained overnight at −80°C. After thawing, 35 µL of 100 mM PMSF and 280 µL of 10 mg/mL HEWL were added and the samples were incubated for 45 min at 37°C under gentle agitation. To cause membrane lysis, IGEPAL was added to a final concentration of 1% (v/v) and the mixture was maintained in ice for 1 h under agitation. Then, 600 µL of 1 mg/mL DNase I and 600 µL of 1 M MgSO_4_ were added and the resulting mixture was incubated at 37°C for 40 min. IBs were separated by centrifugation at 29000×g for 15 min at 4°C. The resulting IBs were washed once with lysis buffer containing 0.5% Triton X-100 and twice with water. After a final centrifugation at 29000×g for 15 min at 4°C, the *pellet* was stored at −80°C and reconstituted in PBS buffer (137.0 mM NaCl, 2.7 mM KCl, 4.3 mM Na_2_HPO_4_, 1.4 mM KH_2_PO_4_, at pH 7.3).

### CR Absorbance

Interaction of CR with IBs was tested using a Jasco V-630 spectrophotometer (Tokyo, Japan) by recording the absorbance spectra from 400 nm to 700 nm using a 10 mm quartz cell. FL TDP-43 IBs, Ct TDP-43 IBs and control IBs at 1.0, 1.0 and 0.7 mg/mL concentrations, respectively, were incubated at 25°C and an aliquot of 60 µL of each sample was mixed with 440 µL of a 5 mM NaH_2_PO_4_, 150 mM NaCl buffer at pH 7.4 containing 20 µM CR. Spectra were then recorded. Spectra were also recorded for similar samples devoid of CR and similar samples devoid of IBs. The difference spectrum obtained by subtracting the spectra of CR alone and IBs alone from that of CR plus IBs indicated the spectrum of CR bound to β-sheet structure. The CR spectra obtained for the native HypF-N protein were also recorded as a further control.

### ThT Fluorescence

FL TDP-43 IBs, Ct TDP-43 IBs and control IBs at the same concentrations described above were incubated at 25°C and an aliquot of 60 µL of each sample was mixed with 440 µL of a 25 mM NaH_2_PO_4_ buffer at pH 6.0 containing 25 µM ThT. The resulting fluorescence was measured at 25°C using a Perkin-Elmer LS 55 spectrofluorimeter (Waltham, MA, USA) equipped with a thermostated cell holder attached to a Haake F8 water bath (Karlsruhe, Germany), using excitation and emission wavelengths of 440 and 450–600 nm, respectively. A 2×10 mm quartz cuvette was used. The ThT spectrum obtained in the presence of the same buffer without IBs was subtracted from those acquired in the presence of FL TDP-43 IBs, Ct TDP-43 IBs and control IBs. The ThT fluorescence obtained after incubation with native HypF-N is reported as a negative control.

### Far-UV CD

FL TDP-43 IBs, Ct TDP-43 IBs and control IBs at 2.8, 2.8 and 2.0 mg/mL concentrations, respectively, were prepared in 25 mM NaH_2_PO_4_ buffer at pH 7.3, 25°C. The far-UV circular dichroism (far-UV CD) spectra were collected over the 190–260 nm wavelength range at 25°C using a Jasco J-810 Spectropolarimeter (Tokyo, Japan) equipped with a thermostated cell holder attached to a Thermo Haake C25P water bath (Karlsruhe, Germany). A 1 mm path-length cell was used. All spectra were blank subtracted. For the calculation of the molar ellipticity [θ] we used the following formula:
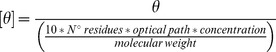
(1)were [θ] is the molar ellipticity in deg cm^2^ dmol^−1^, θ is the ellipticity in mdeg, optical path is in cm, concentration is in g/L, molecular weight is in g/mol.

### FTIR

Purified IBs were resuspended in D_2_O to achieve a final protein concentration of 21 mg/mL for FL TDP-43 IBs and Ct TDP-43 IBs and 15 mg/mL for control IBs. Each sample was deposited on a potassium bromide window in a semipermanent liquid cell using a spacer of 25 µm, and the Fourier transformed infrared spectroscopy (FTIR) spectrum was recorded at room temperature using a Jasco FTIR 4200 spectrophotometer (Tokyo, Japan). The system was constantly purged with N_2_. The resulting spectra were background subtracted and baseline corrected.

### PK Proteolysis

FL TDP-43 IBs and control IBs were prepared in water at a final protein concentration of 14.3 mg/mL and 10.0 mg/mL, respectively, and digested with 250 µg/mL PK at 37°C. The digestion was followed for 500 s observing the turbidity decrease at 350 nm using a Jasco V-630 spectrophotometer (Tokyo, Japan) and a 5 mm quartz cell. The digestion kinetics were analysed using a procedure of best fitting obtained with the following single exponential equation:
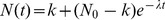
(2)where *N*(*t*) is the turbidimetry at time *t*, *k* is the final value of the turbidimetry, *N*_0_ is the initial turbidimetry, *t* is the time in *s*, *λ* is the rate constant in *s* ^−1^. The FL TDP-43 IBs and Ct TDP-43 IBs digestions were also followed by SDS-PAGE. The FL TDP-43 IBs sample at a total protein concentration of 14.3 mg/mL was treated at 37°C with 250 µg/mL and 20 µg/mL PK, for a total time of 300 s, while the Ct TDP-43 IBs sample at the same concentration as FL TDP-43 was treated at 37°C with 20 µg/mL PK, for a longer time (1200 s). Aliquots of both samples were taken at defined times and tested by SDS-PAGE using 12% (w/v) polyacrylamide gels. The progressive FL TDP-43 and Ct TDP-43 digestions were monitored observing the intensity changes of their corresponding bands.

### AFM

Purified IBs were resuspended in water at a protein concentration of 2 mg/mL and diluted 10 or 100 times. A 10 µL aliquot was deposited on freshly cleaved mica and dried under mild vacuum. Digestion with PK was performed by incubating 40 µL of undiluted IBs with 3.2 µL of a 10 mg/mL PK stock solution for 60 min at 37°C. Digested samples were then diluted 100 times and a 10 µL aliquot was deposited on freshly cleaved mica and dried under mild vacuum. Tapping mode atomic force microscopy (AFM) images were acquired in air using a Dimension 3000 SPM, equipped with a “G” scanning head (maximum scan size of 100 µm) and driven by a Nanoscope IIIa controller, and a Multimode SPM equipped with a “E” scanning head (maximum scan size of 10 µm) and driven by a Nanoscope V controller (Digital Instruments, Veeco). Single-beam uncoated silicon cantilevers (type OMCL-AC160TS, Olympus) were used. The drive frequency was between 260 and 330 kHz; the scan rate was 0.4–0.8 Hz.

### Cell Cultures

Human SH-SY5Y neuroblastoma cells (A.T.C.C., Manassas, VA) were cultured in DMEM, F-12 HAM with 25 mM HEPES and NaHCO_3_ (1∶1) and supplemented with 10% FBS, 1 mM glutamine and antibiotics. Cell cultures were maintained in a 5% CO_2_ humidified atmosphere at 37°C and grown until they reached 80% confluence for a maximum of 20 passages.

### Cell Internalisation

FL TDP-43 IBs were labeled with fluorescein-5-isothiocyanate (5-FITC) using AnaTagTM 5-FITC Microscale Protein Labeling Kit (AnaSpec, San Jose, CA, USA). Transfection of both labeled and unlabeled FL TDP-43 IBs and unlabeled control IBs into SH-SY5Y neuroblastoma cells was performed using a total protein concentration of 5.7 µg/mL and 4.0 µg/mL, respectively, and the PULSin protein delivery reagent (Polyplus-transfection, Illkirch, France). Cells were also transfected with R-phycoerythrin (R-PE), a green fluorescent protein used as a positive control (excitation wavelength was 488 nm). After 3 h transfection, the incubation medium without serum was replaced with fresh complete medium. After washing with PBS, cells treated with unlabeled IBs were counterstained for 10 min with 50 µg/mL Alexa Fluor 633-conjugated wheat germ agglutinin and fixed in 2% (w/v) buffered paraformaldehyde for 10 min at room temperature (20°C). After plasma membrane permeabilisation with a 3% (v/v) glycerol solution for 5 min, the coverslips were incubated for 60 min with 1∶350 diluted rabbit polyclonal anti-TDP-43 antibodies (Sigma-Aldrich) and then for 90 min with 1∶1000 diluted Alexa Fluor 488-conjugated anti-rabbit antibodies incubated at 37°C. Cells were analysed using a Leica TCS SP5 confocal scanning microscope (Leica Microsystems, Mannheim, Germany), equipped with an argon laser source and a Leica Plan Apo 639 oil immersion objective.

The colocalization of FL TDP-43 IBs or phosphorylated TDP-43 with ubiquitin was monitored using 1∶350 rabbit polyclonal anti-TDP-43 antibody or 1∶500 rabbit anti-TDP-43 phosphorylation sites 409/410 (Cosmo Bio Co., Tokio, Japan) for 60 min at 37°C, 1∶150 mouse monoclonal anti-ubiquitin antibodies (Life Technologies, Carlsbad, CA) for 60 min at 37°C, and then with 1∶1000 Alexa Fluor 488-conjugated secondary antibodies (Life Technologies) for 60 min at 37°C and 594-conjugated secondary antibodies (Life Technologies) for 60 min at 37°C.

### MTT Reduction Assay

The toxicity of intracellularly or extracellularly added FL TDP-43 IBs, control IBs and R-PE was assessed on SH-SY5Y cells seeded in 96-well plates, 24 h after cell transfection or addition to the extracellular medium, by the 3-(4,5-dimethylthiazol-2-yl)-2,5-diphenyltetrazolium bromide (MTT) assay as previously described [30]. The final protein concentrations were 4.0 µg/mL for the cell internalisation analysis and ranged from 7.5 to 860 µg/mL for the extracellular analysis (plus 30% for FL TDP-43 IBs). In a set of experiments, cells were treated with either 5 µM MG-132, 10 mM 3-MA or 40 µM chloroquin, which were added immediately after cell transfection with FL TDP-43 IBs and control IBs.

### Measurement of Intracellular ROS and Caspase-3 Activity

The levels of intracellular ROS production and caspase-3 activity were analysed 24 h after transfection or extracellular addition of FL TDP-43 IBs and control IBs to SH-SY5Y cells seeded on glass coverslips and loaded with CM-H_2_DCFDA, as previously described [31] and using FAM-FLICA™ Caspases 3&7 solution (Caspase 3&7 FLICA kit FAM-DEVDFMK, Immunochemistry Technologies, Bloomington, MN) [32]. Final protein concentrations were 4.0 µg/mL and 215 µg/mL (plus 30% for FL TDP-43 IBs) for the intracellular and extracellular analyses, respectively. Aβ_42_ oligomers at a final protein concentration of 60 µg/mL were also used as a positive control. To quantify the signal intensity of CM-H_2_DCFDA, 10–22 cells were analysed using ImageJ software (NIH, Bethesda, MD), and the fluorescence intensities were expressed as arbitrary units.

### Statistical Analysis

Data were expressed as mean ± standard deviation (SD). Comparisons between different groups were performed using ANOVA followed by Bonferroni’s post-comparison test. A p-value lower than 0.05 was considered statistically significant. The single (*), double (**) and triple (***) asterisks refer to p values lower than 0.05, 0.01 and 0.001, respectively.

## Results

### Aggregation of FL TDP-43 and Ct TDP-43 in IBs of *E. coli* Cells

**Figure 1:**
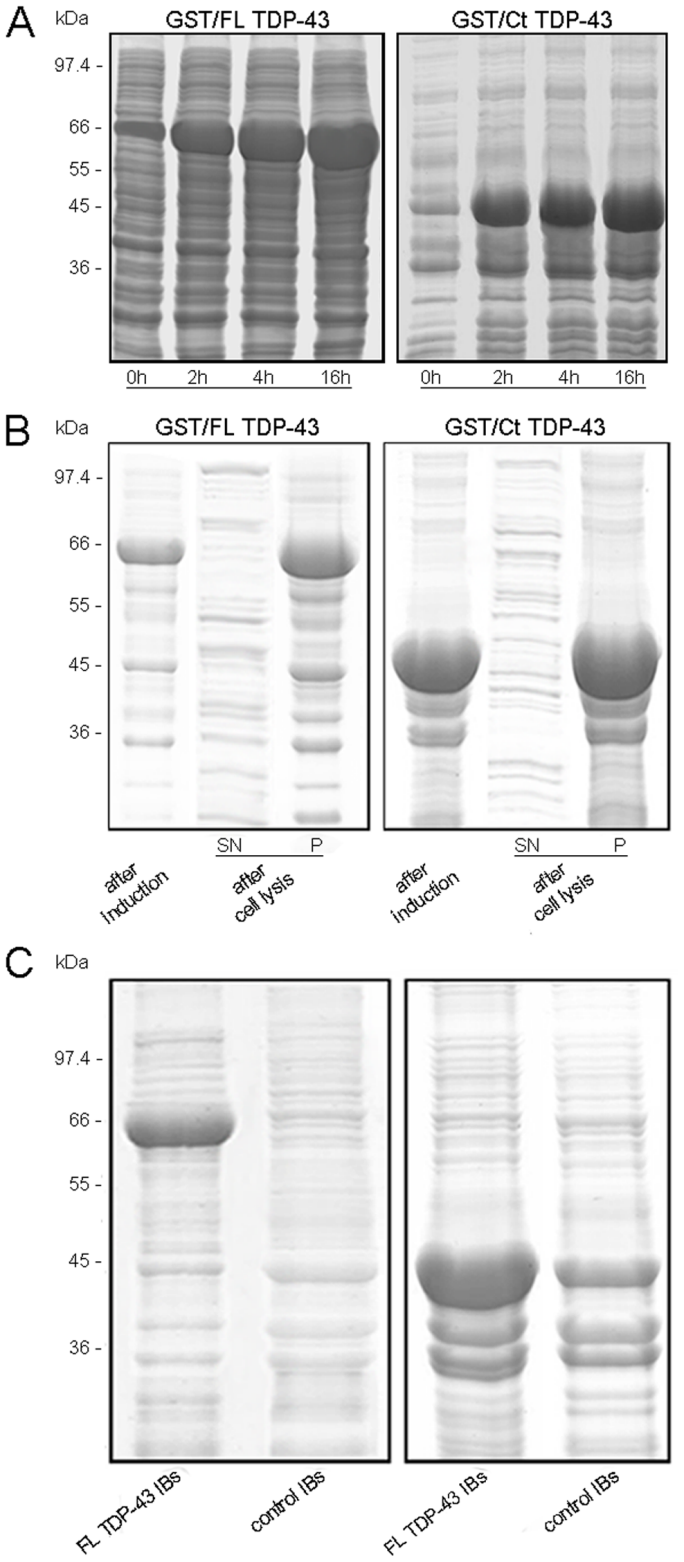
FL and Ct TDP-43 expression in *E. coli* cells. (A) SDS-PAGE analysis of bacterial proteins at different GST/FL TDP-43 (left) and GST/Ct TDP-43 (right) induction times (0 h, 2 h, 4 h and 16 h) with 1 mM IPTG at the temperature of 37°C. The band at ∼ 69 kDa indicates GST/FL TDP-43 (left) and that at ∼ 46 kDa represents GST/Ct TDP-43 (right). (B) SDS-PAGE analysis of total bacterial proteins after GST/FL TDP-43 (left) and GST/Ct TDP-43 (right) inductions with 1 mM IPTG for 16 h at 37°C and of the P and SN fractions of the same samples after cell lysis. (C) SDS-PAGE analysis of purified FL TDP-43 IBs, Ct TDP-43 IBs and control IBs obtained with 1 mM IPTG expression for 16 h at 37°C. The bands at ∼ 69 kDa and ∼ 46 kDa indicate the GST/FL TDP-43 and GST/Ct TDP-43 proteins, respectively, that are absent in the control IBs sample.

The cDNA encoding FL TDP-43 and Ct TDP-43 were cloned downstream of the GST gene in the pGEX-2T plasmid. XL1-Blue cells of *E. coli* were then transformed with the two resulting engineered plasmids. To test whether bacterial cells expressed the fusion GST/FL TDP-43 protein, we evaluated the expression at 37°C with 1 mM IPTG concentration and using SDS-PAGE (Fig. 1A). The GST/FL TDP-43 band corresponds to a molecular weight of ∼ 69 kDa, i.e. the sum of the molecular weights of GST (∼ 26 kDa) and FL TDP-43 (∼ 43 kDa). Immediately before adding IPTG (0 h), a basal protein expression was present. At 2 h and 4 h from induction a greater quantity of expressed protein was evident. However, the major expression of the fusion protein was observed at 16 h. The same conditions were used to evaluate the bacterial expression of the fusion GST/Ct TDP-43 protein. A similar expression level to that of the GST/FL TDP-43 protein was observed by SDS-PAGE, with a band corresponding to a molecular weight of ∼ 46 kDa, that is the sum of the molecular weights of GST (∼ 26 kDa) and Ct TDP-43 (∼ 20 kDa) (Fig. 1A).

After cell growth for 8 h and protein expression for 16 h at 37°C, the bacteria cells were harvested, lysed and centrifuged and the resulting supernatant (SN) and *pellet* (P) were analysed by SDS-PAGE (Fig. 1B). Both GST/FL TDP-43 and GST/Ct TDP-43 were found entirely in the P fraction indicating that they precipitated in IBs (Fig. 1B). Bacterial IBs have been shown to contain amyloid-like aggregates, as determined with CR and ThT binding, FTIR, X-ray diffraction, AFM and transmission electron microscopy (TEM) [25], [26], [28], [33]–[35]. The presence of residual native-like structures and disordered chain segments has also been described, their content depending on the particular IB-forming protein [36]–[40]. Therefore, bacterial IBs are a physiological system consisting of several types of protein aggregates, both amyloid and amorphous, depending on the aggregating proteins. Thus, the aggregation of GST/FL TDP-43 and GST/Ct TDP-43 into IBs offered the possibility of investigating whether full-length TDP-43 or its C-terminal fragment have the propensity to form amyloid or other forms of self-assembly when aggregating in the highly crowded environment existing in a living organism.

The IBs formed from cells expressing GST/FL TDP-43 (FL TDP-43 IBs), GST/Ct TDP-43 (Ct TDP-43 IBs) and GST (control IBs) were purified as described in *Materials and Methods.* Using a densitometric analysis of the SDS-PAGE bands we evaluated that the GST/FL TDP-43 band was 29±3% of all proteins present in FL TDP-43 IBs (Fig. 1C), while the GST/Ct TDP-43 band was 32±2.9% of all proteins present in Ct TDP-43 IBs (Fig. 1C). Therefore, for all the biophysical data present in this work, we analysed TDP-43 IBs with a total protein concentration higher than that present in control IBs by ∼ 30%, so that the three IBs samples contained the same amount of proteins distinct from TDP-43. Using this approach, it was possible to evaluate the contribution of TDP-43 relative to other proteins present in IBs by difference, for example by subtracting the FTIR spectrum of control IBs from that of FL TDP-43 IBs or from that of Ct TDP-43 IBs.

### FL TDP-43 and Ct TDP-43 Aggregates do not Bind CR and ThT

**Figure 2:**
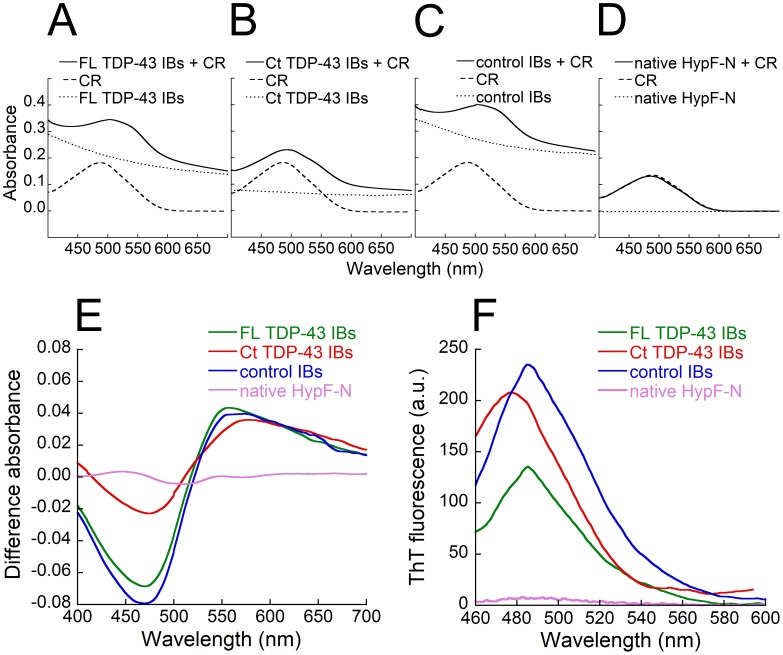
CR and ThT binding of FL TDP-43 IBs, Ct TDP-43 IBs and control IBs. (A) Absorbance spectra of FL TDP-43 IBs+CR (solid line), CR (dashed line) and FL TDP-43 IBs (dotted line). (B) Absorbance spectra of Ct TDP-43 IBs+CR (solid line), CR (dashed line) and Ct TDP-43 IBs (dotted line). (C) Absorbance spectra of control IBs+CR (solid line), CR (dashed line) and control IBs (dotted line). (D) Absorbance spectra of native HypF-N+CR (solid line), CR (dashed line) and native HypF-N (dotted line). (E) Difference absorbance spectra obtained for FL TDP-43 IBs (green), Ct TDP-43 IBs (red), control IBs (blue) and native HypF-N (purple). (F) ThT fluorescence spectra in the presence of FL TDP-43 IBs (green), Ct TDP-43 IBs (red), control IBs (blue) and native HypF-N (purple).

We used the amyloid diagnostic CR dye to assess whether FL TDP-43 and Ct TDP-43 contained in IBs display typical amyloid properties. The CR absorbance increased in the presence of FL TDP-43 IBs and the wavelength of maximum absorption red-shifted to ∼ 508 nm (Fig. 2A). This spectral change was very similar to that observed in the presence of control IBs (Fig. 2C). The CR absorbance increased also in the presence of Ct TDP-43 IBs, although less markedly than in the presence of FL TDP-43 IBs, with a maximum absorption at ∼ 495 nm (Fig. 2B). Spectral changes of CR were not observed in the presence of native HypF-N, used here as a soluble protein and, thus, as a negative control (Fig. 2D). For the three IBs samples, the difference spectrum obtained subtracting the CR spectrum and the IBs spectrum from the CR spectrum in the presence of IBs, showed a characteristic peak at ∼ 550 nm, typical of CR bound to amyloid aggregates (Fig. 2E). By contrast, the difference spectrum obtained by subtracting the CR spectrum and the native HypF-N spectrum from the CR spectrum in the presence of native HypF-N was flat (Fig. 2E). The observation that the difference spectra obtained with FL TDP-43 IBs, Ct TDP-43 IBs and control IBs are superimposable at ∼550 nm, indicates that TDP-43 does not seem to contribute to the amyloid-like structures present in IBs.

We then analysed the capacity of the same samples to bind the ThT dye and increase its fluorescence. FL TDP-43 IBs increased the ThT fluorescence less markedly than control IBs, although this difference was not statistically significant (p>0.05); moreover, Ct TDP-43 increased the ThT fluorescence similarly to control IBs (Fig. 2F). Hence, the ThT assay confirms that the FL and Ct TDP-43 aggregates present in IBs do not have an amyloid-like structure (Fig. 2F). As expected, no ThT fluorescence increase was observed in the presence of non-aggregated native HypF-N (Fig. 2F).

As a positive control for *E. coli* IBs containing amyloid-like aggregates, we purified IBs formed after expression of the second acylphosphatase from *D. melanogaster* (AcPDro2), a protein previously shown to form amyloid-like fibrils *in vitro* [41]. In particular, we purified IBs formed after expression of the destabilised C43S mutant of the protein (C43S AcPDro2 IBs), since the wild-type protein was soluble after expression and the resulting IBs contained little protein (wt AcPDro2 IBs). Unlike the wt AcPDro2 IBs, the C43S AcPDro2 IBs were found to bind CR, with a maximum at ∼ 550 nm in the difference spectrum, and to bind ThT, with a remarkable increase of its fluorescence (Fig. S1).

### FL TDP-43 and Ct TDP-43 Aggregates are Composed of a Random Coil Structure

**Figure 3:**
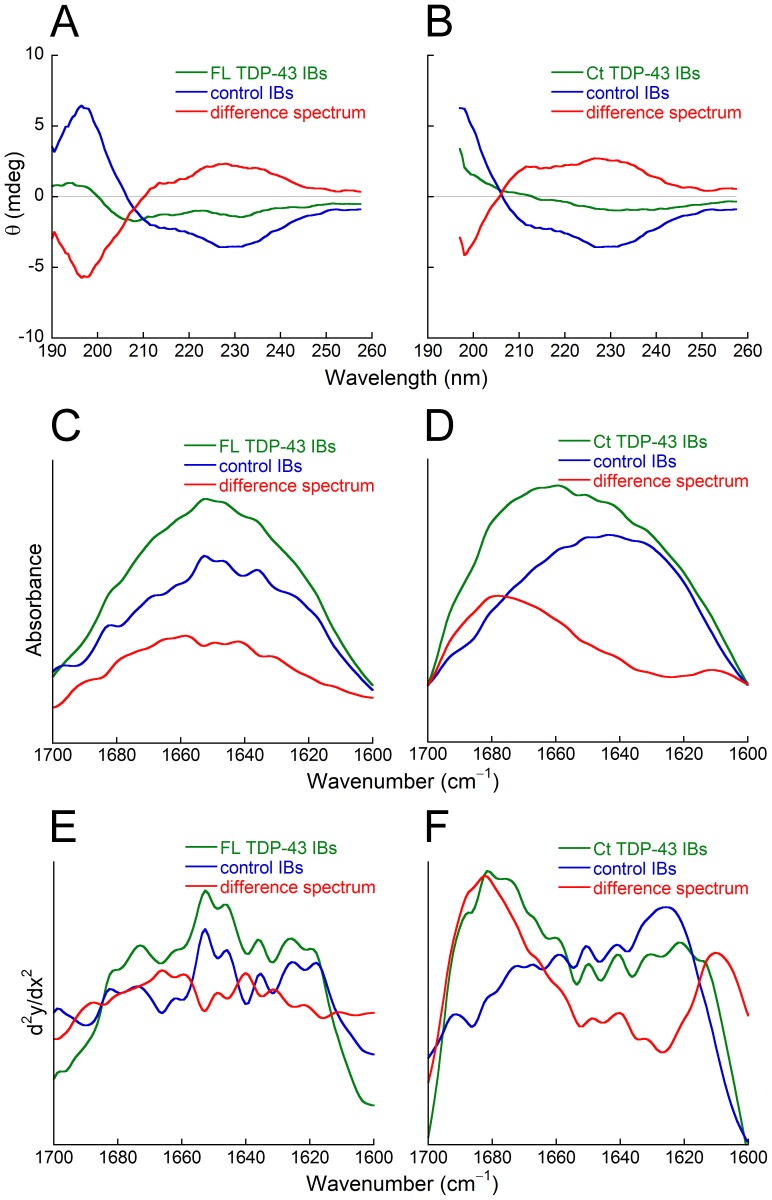
Secondary structure analysis of FL TDP-43 IBs, Ct TDP-43 IBs and control IBs. CD spectra (A,B), amide I regions of FTIR spectra (C,D) and second derivative of FTIR spectra (E,F) of FL TDP-43 IBs (A,C,E) and Ct TDP-43 IBs (B,D,F). In both analyses, the spectra are those of TDP-43 IBs (green), control IBs (blue) and difference spectra obtained subtracting the latter from the former (red).

Amyloid fibrils are closely associated with a β-sheet content that can be typically detected with either far-UV CD, FTIR or X-ray fiber diffraction. The CD spectra obtained in the presence of FL TDP-43 IBs and control IBs display a negative peak at ca. 220–230 nm and a positive peak at ca. 190–200 nm (Fig. 3A), which is typical of large aggregates containing amyloid fibrils [42], [43]. The difference spectrum between them discloses the secondary structure of FL TDP-43 aggregates wherein the presence of a random coil structure is evident, as shown by a negative peak at ∼ 198 nm (Fig. 3A). A similar result was obtained with Ct TDP-43 IBs and control IBs, with the CD spectra displaying a negative peak at ∼ 230 nm and a positive peak below 197 nm (Fig. 3B). Similarly to the result obtained with FL TDP-43 aggregates, a largely random coil structure emerges from the difference spectrum, with a negative peak at ca. 198 nm (Fig. 3B).

The Amide I regions of FTIR spectra of FL TDP-43 IBs and control IBs obtained in D_2_O, as well as their second derivative spectra, display a prominent peak at ∼ 1650 cm^−1^ corresponding to α-helix and unordered structures, another peak at ∼ 1675 cm^−1^ corresponding to β-turn structure and the characteristic peak at ∼ 1625 cm^−1^ associated with the presence of intermolecular β-sheet structures (Fig. 3C,E). The difference spectrum obtained from them shows two major bands at ∼ 1667 cm^−1^, corresponding to β-turn structure, and ∼ 1640 cm^−1^, corresponding to unordered structure for an FTIR spectrum obtained in D_2_O (Fig. 3C,E). Importantly, the difference spectrum shows a clear disappearance of the peak in the intermolecular β-sheet region (Fig. 3C,E), providing further evidence for the lack of β-sheet structure and presence of disordered structure in the FL TDP-43 aggregates present in IBs.

The Amide I region of the FTIR spectrum of Ct TDP-43 IBs in D_2_O and its second derivative, along with those of control IBs obtained concomitantly, display a similar peak at ∼ 1650 cm^−1^ associated with α-helix and unordered structures (Fig. 3D,F). Besides, Ct TDP-43 IBs spectra show a prominent peak at ∼ 1680 cm^−1^ associated with β-turn structure and a secondary peak at ∼ 1620 cm^−1^ corresponding to intermolecular β-sheet structures, while control IBs spectra show the major peak at ∼ 1620 cm^−1^ indicating the presence of intermolecular β-sheet structures and a smaller peak at ∼ 1670 cm^−1^ associated with β-turn structure (Fig. 3D,F). The difference spectrum obtained from them shows a major peak at ∼ 1680 cm^−1^ associated with β-turn structure and another smaller peak at ∼ 1645 cm^−1^ corresponding to unordered structure (Fig. 3D,F). Again, the difference spectrum shows no peaks in the intermolecular β-sheet region, indicating a largely disordered structure in the Ct TDP-43 aggregates present in IBs.

The amide I region of the FTIR spectrum obtained with C43S AcPDro2 IBs, used here as a positive control for bacterial IBs containing amyloid-like aggregates, features a remarkable peak in the β-sheet region, unlike the wt AcPDro2 IBs, indicating the presence of a largely β-sheet structure in the C43S AcPDro2 aggregates (Fig. S2).

### PK Digests FL TDP-43 and Ct TDP-43 Aggregates Contained in IBs

**Figure 4:**
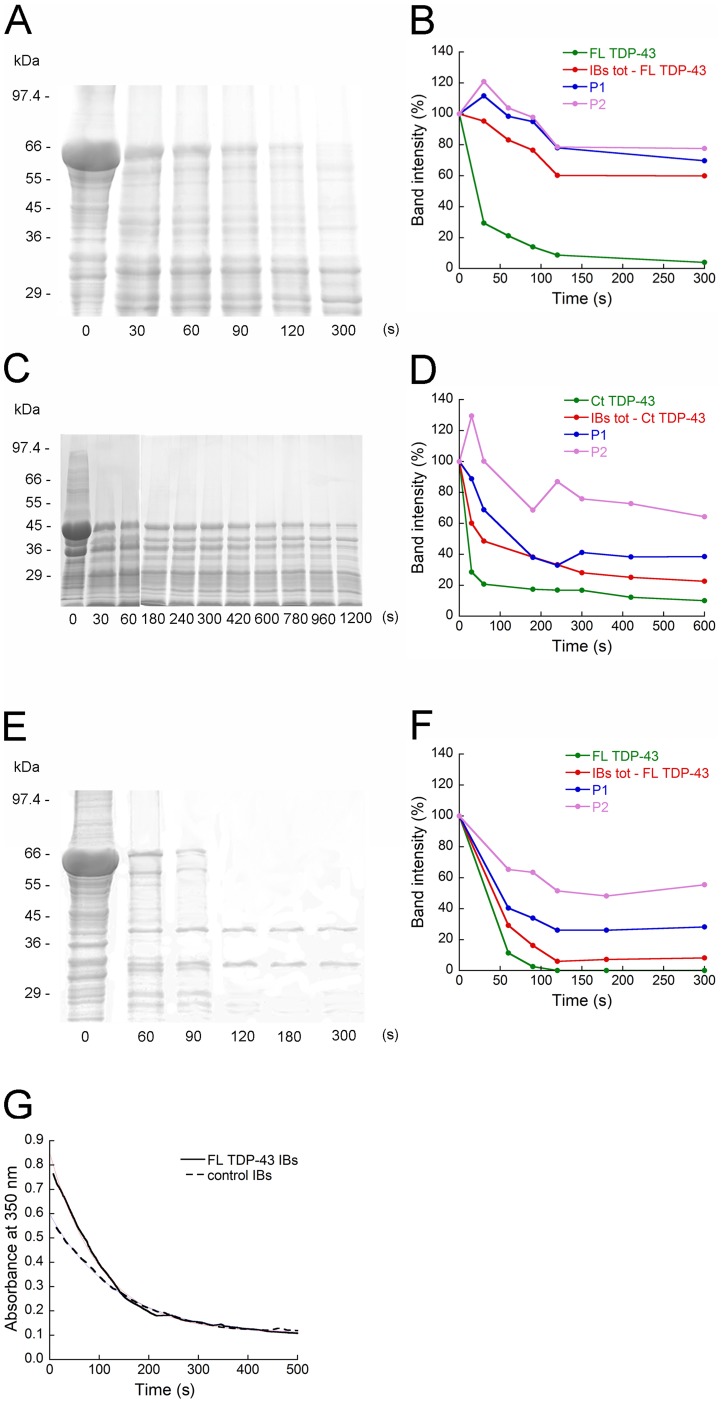
PK proteolysis of FL TDP-43 IBs, Ct TDP-43 IBs and control IBs. (A) SDS-PAGE of FL TDP-43 IBs after incubation with PK at the indicated times. The temperature was 37°C, the total protein concentration of FL TDP-43 IBs was 14.3 mg/mL and the PK concentration was 20 µg/mL. The band at ∼ 69 kDa indicates GST/FL TDP-43. (B) Corresponding densitometric analysis graph of SDS-PAGE bands representing FL TDP-43 (green), proteins other than FL TDP-43 (red) and two *E. coli* proteins labeled P1 and P2 (blue and purple). (C) SDS-PAGE of Ct TDP-43 IBs after incubation with PK at the indicated times. Conditions were as in panel A. The band at ∼ 46 kDa indicates GST/Ct TDP-43. (D) Corresponding densitometric analysis graph of SDS-PAGE bands representing Ct TDP-43 (green), proteins other than Ct TDP-43 (red) and two *E. coli* proteins labeled P1 and P2 (blue and purple). (E) SDS-PAGE of FL TDP-43 IBs after incubation with PK at the indicated times. The temperature was 37°C, the total protein concentration of FL TDP-43 IBs was 14.3 mg/mL and the PK concentration was 250 µg/mL. (F) Corresponding densitometric analysis graph of SDS-PAGE bands representing FL TDP-43 (green), proteins other than FL TDP-43 (red) and two *E. coli* proteins labeled P1 and P2 (blue and purple). (G) Kinetics at 37°C of PK digestion of FL TDP-43 IBs and control IBs monitored by turbidimetry at 350 nm. The total protein concentration for FL TDP-43 IBs and control IBs was 14.3 mg/mL and 10 mg/mL, respectively, and the PK concentration was 250 µg/mL.

PK is a protease usually used to map the protected core of amyloid fibrils because it is highly active against peptide bonds of globular and disordered proteins, but it cannot attack the highly packed backbones in amyloid β-sheet structures [44]. Both FL TDP-43 IBs and Ct TDP-43 IBs were treated with 20 µg/mL of PK and their protein content was analysed using SDS-PAGE. At this PK concentration a fast cleavage of FL TDP-43 was observed, with its band disappearing after 5 min of incubation (Fig. 4A,B). A fast cleavage was also observed for Ct TDP-43 (Fig. 4C,D). In this case the Ct TDP-43 band does not seem to disappear completely after 20 min of incubation, but it is reasonable to assume that the apparent remaining band was associated with an *E. coli* protein present in IBs having a molecular weight superimposed to that of Ct TDP-43 (compare Ct TDP-43 IBs and control IBs in Fig. 1C). FL TDP-43 IBs were also treated with a higher PK concentration (250 µg/mL), which induced a rapid disappearance after only 120 s (Fig. 4E,F). Other *E. coli* proteins present in TDP-43 IBs were susceptible to the action of PK, but they were generally more resistant, indicating the presence of amyloid-like structures in the IBs (Fig. 4A–F). Thus, these data confirm further the absence of an amyloid core in the FL TDP-43 and Ct TDP-43 aggregates contained in IBs.

The higher PK concentration (250 µg/mL) was also utilized for treating both FL TDP-43 IBs and control IBs and follow the decrease in the turbidimetry signal at 350 nm (Fig. 4G). At time zero the turbidimetry caused by FL TDP-43 IBs was greater than that of control IBs, because of the 30% protein concentration difference present in FL TDP-43 IBs relative to control IBs. A time dependent decrease in the absorbance value was found to occur for both FL TDP-43 IBs and control IBs, due to digestion of non-amyloid aggregates. After 140 s the two curves were apparently superimposable and reached gradually a plateau. This implies that the FL TDP-43 aggregates were entirely digested by PK within 140 s, confirming the absence of a PK-resistant cross-β organization in these aggregates.

### FL TDP-43 IBs, Ct TDP-43 IBs and Control IBs Appear Morphologically Irregular

**Figure 5:**
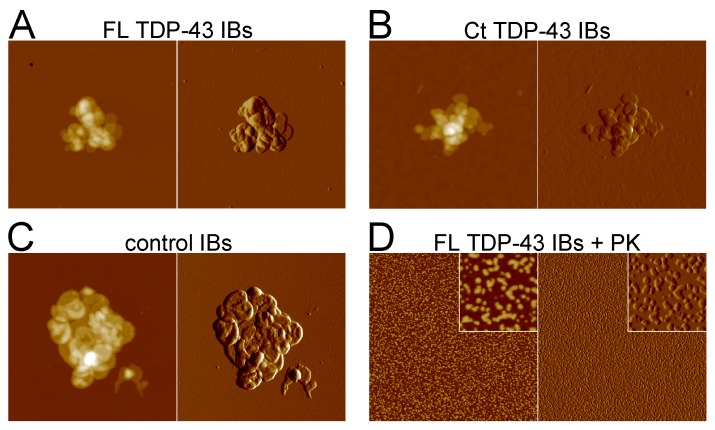
Morphology of FL TDP-43 IBs, Ct TDP-43 IBs and control IBs observed with AFM. (A–C) FL TDP-43 IBs, Ct TDP-43 IBs and control IBs, respectively. The scan size is 2 µm. (D) FL TDP-43 IBs after 1 h incubation with 20 µg/mL PK. The scan size is 2 µm. The inset shows a detail of the main panel at higher magnification (scan size 250 nm). In all panels left and right images represent height and amplitude data, respectively. Z range: 130 nm (A), 100 nm (B), 90 nm (C), 10 nm (D).

AFM was used to investigate the morphology of FL TDP-43 IBs, Ct TDP-43 IBs and control IBs. All IBs samples appeared as irregular structures with heights of 33±2 nm for FL TDP-43 IBs (Fig. 5A), 36±3 nm for Ct TDP-43 IBs (Fig. 5B) and 32±2 nm for control IBs (Fig. 5C). Such structures were formed by a variable number of disk or crescent-shaped subunits having a height of 7–8 nm (Fig. 5A–C). After 1 h digestion with 20 µg/mL PK all IBs were proteolysed and only a large number of small structures having a height of ∼ 3 nm remained in the samples (as exampled by FL TDP-43 IBs digestion reported in Fig. 5D), possibly representing small aggregates of the lipids and polypeptides that are known to be present in bacterial IBs [45]–[47]. Interestingly, the absence of fibrillar structures after PK treatment indicates that the TDP-43 aggregates present in the IBs are not amyloid-like. Moreover, although the ThT and CR assays, as well as CD and FTIR spectra, show the existence of amyloid aggregates in both FL TDP-43 IBs and Ct TDP-43 IBs and arising from non-TDP-43 proteins, AFM imaging after PK digestion reveals that such structures are unstable and less packed and organized then the known amyloid aggregates.

### Transfected FL TDP-43 IBs are Toxic to Cultured Neuronal Cells

**Figure 6:**
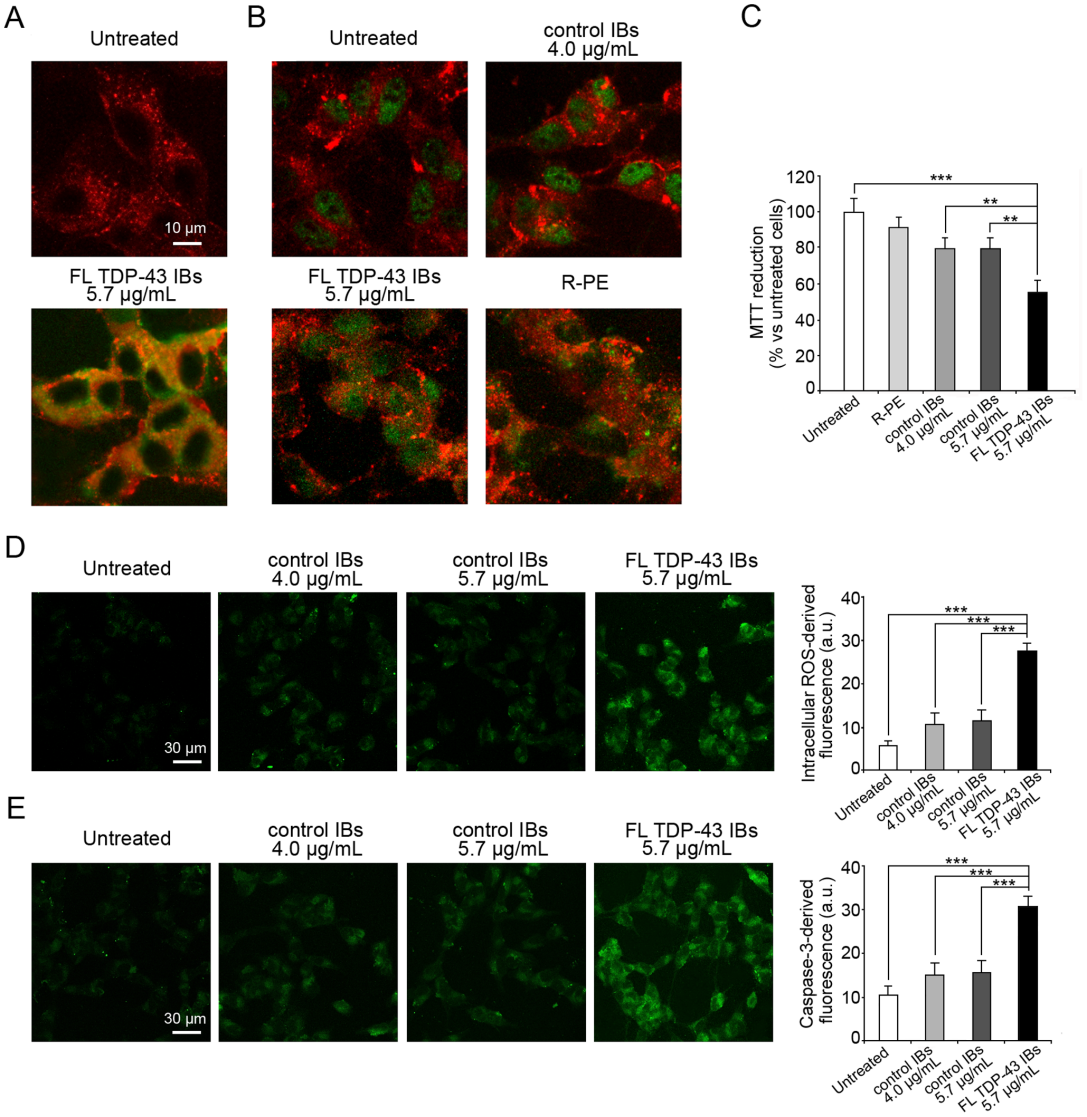
Toxicity of FL TDP-43 IBs and control IBs delivered intracellularly. (A) Representative confocal scanning microscope images of SH-SY5Y cells transfected with FL TDP-43 IBs labeled with 5-FITC. Red and green fluorescences indicate cell profiles and labeled FL TDP-43 IBs, respectively; the images were analysed at median planes parallel to the coverslip. (B) Representative confocal scanning microscope images of SH-SY5Y cells transfected with FL TDP-43 IBs, control IBs and R-PE. Red fluorescence indicates cells profiles and green fluorescence indicates TDP-43 detected with immunofluorescence or R-PE. (C) MTT reduction of SH-SY5Y cells following transfection with FL TDP-43 IBs (5.7 µg/mL), control IBs (4.0 µg/mL and 5.7 µg/mL) and R-PE (4.0 µg/mL). (D,E) Representative confocal scanning microscope images of SH-SY5Y cells showing intracellular ROS levels (D) and caspase-3 activation (E) after transfection with FL TDP-43 IBs (5.7 µg/mL) and control IBs (4.0 µg/mL and 5.7 µg/mL). The green fluorescence arises from the CM-H_2_DCFDA probe that has reacted with ROS and from FAM-FLICA™ Caspase 3&7. The histograms show the quantitative fluorescence values in both cases. In panels C-E untreated cell means cells treated with the transfection mix only without proteins. The double (**) and triple (***) asterisks refer to p values lower than 0.01 and 0.001, respectively.

FL TDP-43 IBs and control IBs were also tested for their ability to cause cellular dysfunction in cultured neuroblastoma SH-SY5Y cells. Both forms of IBs were used to transfect the cells by means of the PULSin protein delivery reagent (Polyplus-transfection, Illkirch, France), which contains a cationic amphiphilic molecule thus facilitating the entry of the IBs in the cellular cytoplasm. In a first experiment, carried out to verify directly the presence of the FL TDP-43 IBs inside the cytoplasm following transfection, FL TDP-43 IBs were labeled with 5-FITC and then used to transfect the cells. The images obtained with confocal microscopy show the presence of abundant exogenous TDP-43 aggregates in the cytoplasm, indicating the high yield of FL TDP-43 IBs transfection (Fig. 6A).

In another set of experiments, both FL TDP-43 IBs and control IBs were used to transfect the cells and the presence of FL TDP-43 in the transfected cells was assessed using anti-TDP-43 polyclonal antibodies. Untreated (non-transfected) cells showed the presence of abundant TDP-43 in the nuclei, as expected for cells expressing endogenous TDP-43 which has a nuclear localization signal (Fig. 6B). Cells transfected with control IBs showed a similar high abundance of endogenous TDP-43 in the nuclei, whereas cells transfected with FL TDP-43 IBs showed a combination of nuclear endogenous TDP-43 and exogenous cytoplasmic TDP-43 (Fig. 6B). Cells were also transfected with R-PE, a green fluorescent protein used as a positive control and indeed shown to be present abundantly in the cytoplasm following transfection (Fig. 6B).

The viability of SH-SY5Y cells treated with FL TDP-43 IBs and control IBs was assessed by measuring their ability to reduce MTT, their levels of intracellular ROS and their caspase-3 activity. All such tests are widely used to assess the toxicity of TDP-43 expressed in eukaryotic cell cultures [21], [48], [49]. In all such tests, cells treated with control IBs were found to be less viable than untreated cells, which are cells treated with the transfection mix only (Fig. 6C–E). However, a significantly lower level of viability was found in cells treated with FL TDP-43 IBs, showing that the presence of FL TDP-43 in the IBs increases their toxicity (Fig. 6C–E). Importantly, the FL TDP-43 concentration was 1.7 µg/mL in the samples used to transfect the cells in all such tests, indicating that the protein aggregated in the non-amyloid form described above is highly toxic.

For the reasons explained above, the FL TDP-43 IBs contained a total protein concentration higher by ca. 30% (5.7 µg/mL) than that of control IBs (4.0 µg/mL). To assess whether the higher toxicity observed for FL-TDP-43 IBs was due to its higher protein content, the cells were also treated with control IBs containing a total protein quantity similar to that of FL TDP-43 IBs (5.7 µg/mL), but the FL-TDP-43 IBs still maintained a significantly higher toxicity with all probes of cell viability (Fig. 6C–E). All the values of toxicity reported here refer to cells transfected with the transfection mix only, ruling out that the observed toxicity of internalised FL TDP-43 IBs originates from the transfection procedure. Moreover, R-PE was not found to decrease significantly the MTT reduction following transfection, ruling out that transfected protein samples are *per se* toxic (Fig. 6C).

### Transfected FL TDP-43 IBs are Partially Ubiquitinated and Phosphorylated in Cultured Neuronal Cells

**Figure 7:**
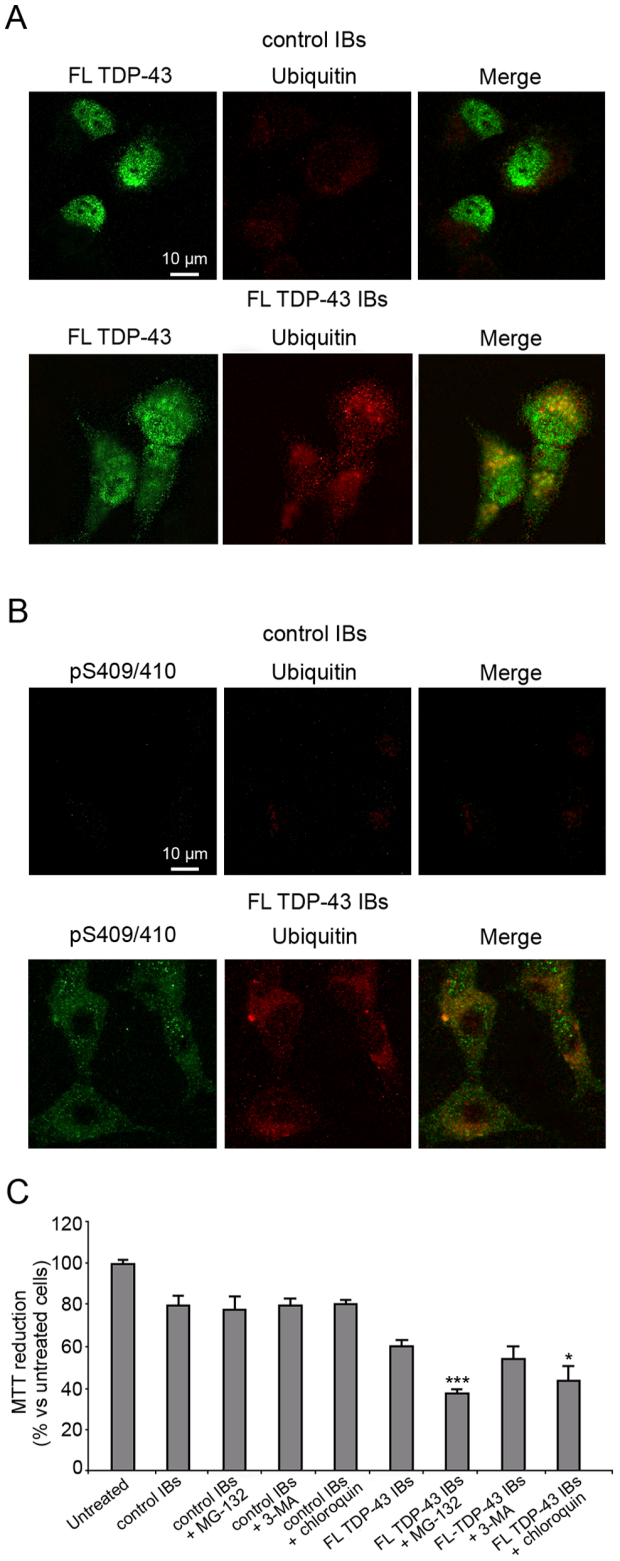
Ubiquitination and phosphorylation of FL TDP-43 IBs and control IBs delivered intracellularly. (A) Representative confocal scanning microscope images showing partial colocalization of exogenous cytoplasmic TDP-43 with ubiquitin-positive aggregates in SH-SY5Y cells transfected with control IBs and FL TDP-43 IBs. Green and red fluorescences indicate TDP-43 and ubiquitin, respectively. (B) Representative confocal scanning microscope images showing partial colocalization of phosphorylated S409/410 of TDP-43 and ubiquitin-positive aggregates in SH-SY5Y cells transfected with control IBs and FL TDP-43 IBs. Green and red fluorescences indicate pS409/410 TDP-43 and ubiquitin, respectively. (C) MTT reduction of SH-SY5Y cells following transfection with FL TDP-43 IBs (5.7 µg/mL), control IBs (4.0 µg/mL) in the absence or presence of 5 µM MG-132, 10 mM 3-MA and 40 µM chloroquin. The single (*) and triple (***) asterisks refer to p values lower than 0.05 and 0.001, respectively.

In order to assess whether the exogenous cytoplasmic FL TDP-43 IBs were ubiquitinated, we analysed the colocalization of FL TDP-43 IBs with ubiquitin. The images obtained with confocal microscopy showed a weak and diffuse cytoplasmic ubiquitin staining in the cytoplasm of SH-SY5Y cells transfected with control IBs, whereas a marked staining and the presence of inclusions was evident in cells transfected with FL TDP-43 IBs (Fig. 7A). In particular, a partial colocalization between cytoplasmic TDP-43 IBs and ubiquitin was observed (Fig. 7A). To investigate whether the exogenous cytoplasmic FL TDP-43 IBs were phosphorylated, we employed antibodies that recognize phosphorylated S409/410 in TDP-43 (Fig. 7B). The confocal images showed a clear phosphorylation of FL TDP-43 IBs (Fig. 7B) and these phosphorylated sites appeared to partially colocalize with ubiquitin (Fig. 7B). All these data suggest that FL TDP-43 IBs are partially ubiquitinated and phosphorylated, recapitulating major features of the endogenous cytoplasmic inclusions found in ALS and FTLD-U.

The toxicity of FL TDP-43 IBs and control IBs was also assessed in the presence of specific inhibitors of the proteasome, authophagy and lisosomes systems, such as MG-132, 3-MA and chloroquin, respectively (Fig. 7C). Cells treated with control IBs in the presence of the three inhibitors were found to decrease MTT reduction to a level similar to control IBs alone (Fig. 7C). Interestingly, a significantly lower level of viability was found in cells treated with FL TDP-43 IBs in the presence of MG-132 with respect to cells treated with FL TDP-43 IBs alone, showing that the inhibition of the proteasome system increases their toxicity (Fig. 7C). The cells treated with FL TDP-43 IBs in the presence of chloroquin also showed a significant decrease of cell viability with respect to cells treated with FL TDP-43 IBs alone, whereas cell treatment with FL TDP-43 IBs in the presence of 3-MA was not found to modify cell viability to a significant extent (Fig. 7C). These data indicate that the TDP-43 aggregates contained in FL TDP-43 IBs are toxic to the transfected cells, which react to their presence via mechanisms dedicated to the clearance of misfolded proteins.

### Extracellular FL TDP-43 IBs are Toxic to Cultured Neuronal Cells

**Figure 8:**
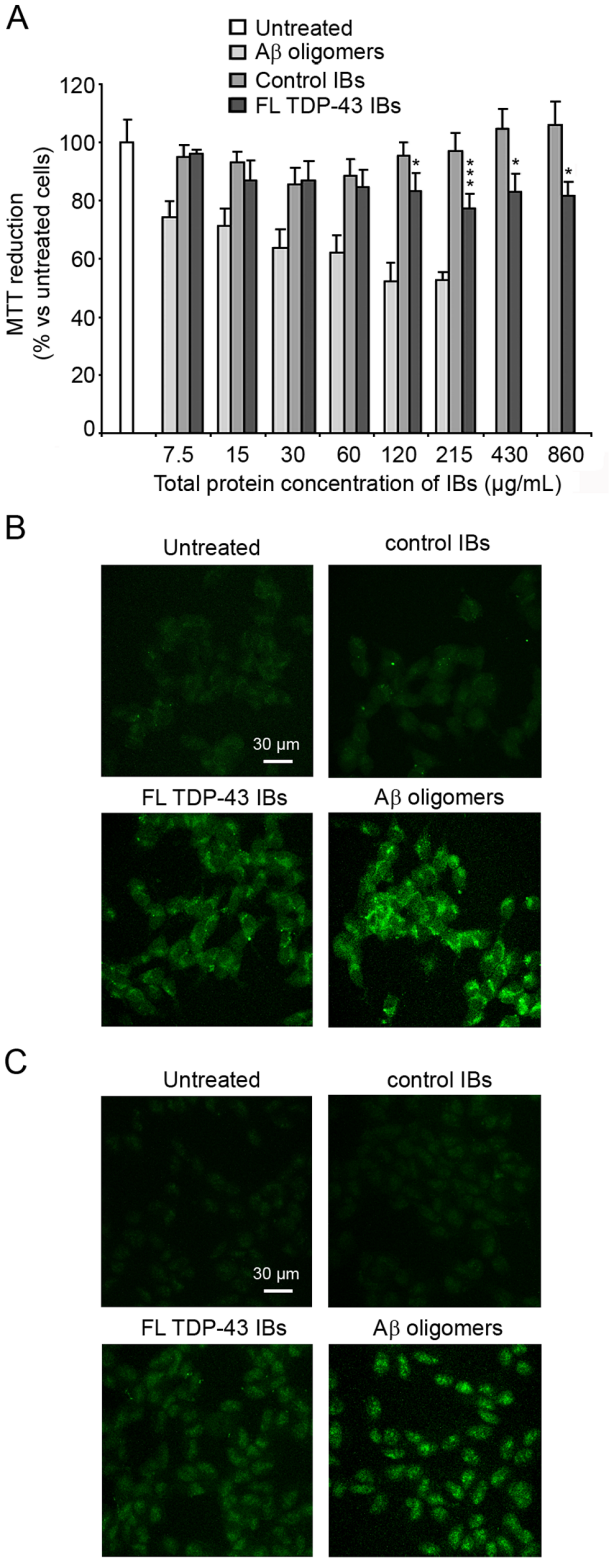
Toxicity of FL TDP-43 IBs and control IBs added extracellularly. (A) MTT reduction of SH-SY5Y cells following extracellular addition of FL TDP-43 IBs, control IBs and Aβ_42_ oligomers. The indicated protein concentration refers to control IBs and corresponds to values higher by 30% for FL TDP-43. The single (*) and triple (***) asterisks indicate a statistically significant difference between FL TDP-43 IBs and untreated cells (p<0.05 and p<0.001, respectively). (B,C) Representative confocal scanning microscope images of SH-SY5Y cells showing intracellular ROS levels (B) and caspase-3 activation (C) after extracellular addition of FL TDP-43 IBs, control IBs and Aβ_42_ oligomers. The green fluorescence arises from the CM-H_2_DCFDA probe that has reacted with ROS and from FAM-FLICA™ Caspase 3&7. Total protein concentrations were 280, 215 and 60 µg/mL for FL TDP-43 IBs, control IBs and Aβ_42_ oligomers, respectively.

The toxicity of FL TDP-43 IBs and control IBs was also assessed by adding them to the extracellular medium of the SH-SY5Y cells in the absence of any transfection procedure. We first evaluated the toxic effect of both forms of IBs by measuring the ability of treated cells to reduce MTT (Fig. 8A). Their toxic effect was analysed at different protein concentrations, ranging from 7.5 µg/mL to 860 µg/mL (plus 30% for FL TDP-43 IBs). Aβ_42_ oligomers were also used as a positive control of toxicity in a range of concentrations varying from 7.5 µg/mL to 215 µg/mL (Fig. 8A). It was found that FL TDP-43 IBs, unlike control IBs, were able to decrease MTT reduction at concentrations of 171 µg/mL or higher (Fig. 8A). FL TDP-43 IBs were also found to cause an increase of intracellular ROS production and caspase-3 activation, whereas control IBs induced only small changes (Fig. 8B,C).

## Discussion

In 2006 it was reported, for the first time, that ubiquitin-positive, tau- and α-synuclein-negative intracellular inclusions found in the spinal cord motor neurons, in the hippocampus and neocortex of sporadic ALS and FTLD-U patients, contained the previously unidentified TDP-43 protein or its C-terminal fragments [4]. Such inclusions appear to be phosphorylated and ubiquitinated [4], [6], [7], but the structure adopted by TDP-43 in such deposits is currently a matter of debate. The IBs formed in *E. coli* cells following the over-expression of TDP-43 are informative in this regard, as bacterial IBs have been shown to consist mainly of amyloid-like deposits, providing an opportunity to emphasize the propensity of this protein to form amorphous aggregates if the amyloid form is not found in the TDP-43 component of the IBs. Our results indicate that both FL and Ct TDP-43 aggregates present in *E. coli* IBs do not possess any of the hallmarks of amyloid fibrils, allowing them to be classified as amorphous. CD and FTIR both show the presence of a secondary random-coil and β-turn structure in the absence of any detectable β-sheet structure. The aggregates bind neither ThT nor CR and are highly susceptible to PK digestion. The AFM analysis does not allow to visualize the morphology of the TDP-43 aggregates within the IBs. However, the IBs containing both FL and Ct TDP-43 appear to have an irregular morphology like that of the control IBs devoid of TDP-43.

The question naturally arises as to whether our findings resolve the current dispute on the structural/morphological type of inclusions present in TDP-43 pathology or rather add one more contribution to the ongoing debate. In order to address this issue we can try to survey the current literature critically. The first reports aimed at characterizing the structure/morphology of TDP-43 inclusions accumulating in ALS and FTLD-U patients have excluded the presence of amyloid-like structures. Although the ultrastructure of TDP-43 inclusions analysed with TEM have highlighted the presence of 10–20 nm filaments [9], [10], [13]–[15], the aggregates were found to be unable to bind ThT, ThS or CR, ruling out the presence of an amyloid-like structure [11], [16]. A very recent report has emphasized that TDP-43 inclusions may consist of amyloid fibrils, following the observation of a few ThS-positive inclusions [13]. Nevertheless, the ThS positivity was found only in a small fraction of skein-like inclusions of the spinal cord; it was indeed absent in most spinal cord skeins and absent altogether in other types of TDP-43 inclusions of the spinal cord [13]. All inclusions of the brain were also ThS negative [13]. By contrast, the most recent paper reporting a diffuse ThS positivity in all TDP-43 inclusions analysed in ALS spinal cords and FTLD-U brains raises important questions on the results obtained previously [12]. This study originated from a chemically harsh treatment of the tissue sections isolated from ALS and FTLD-U patients, based on the sequential use of potent oxidants, reductants, acids and bases, such as permanganate, metabisulfite, oxalic acid, sodium hydroxide and hydrogen peroxide, all known to chemically modify proteins and hydrolyse peptide bonds. It cannot be ruled out that the biological inclusions undergo a heavy structural reorganization under such circumstances.

The difficulty of purifying TDP-43 has limited the study of TDP-43 aggregation *in vitro* and is likely to be a limiting factor in future research on TDP-43. One report exists, however, on the characterization of the aggregates formed *in vitro* from TDP-43 after its purification [18]. This study has confirmed a filamentous morphology of TDP-43 aggregates, in the absence of ThT and CR binding [18]. By contrast, four other reports have emphasized an amyloid-like structure of TDP-43 aggregates, but all of them have referred to short peptides of 13 to 50 residues, all obtained from the sequence of TDP-43 [19]–[22]. It is well known that protein fragments have generally aggregation properties different from those of the full-length protein and it was recently reported that amyloid-like aggregation, as opposed to structurally undefined aggregation, is favored by small peptides and proteins [50].

Hence, our data and analysis suggest that FL and Ct TDP-43 form amorphous aggregates rather than amyloid-like. The finding that most, if not all, TDP-43 aggregates are amorphous in the spinal cord and brain of ALS and FTLD-U patients, in bacterial IBs and finally in aggregates formed *in vitro* from purified TDP-43, suggests that this protein has an intrinsic propensity to form amorphous aggregates and that such a propensity is not affected remarkably by specific factors present in the biological compartments where aggregation occurs.

The bacterial TDP-43 IBs characterized here were found to be highly toxic to cultured neuronal cells, particularly following their internalization in the cytoplasm, where they are at least in part ubiquitinated and phosphorylated. A significant toxicity was found using intracellularly delivered IBs where TDP-43 was present at a concentration as low as 1.7 µg/mL before internalisation. It is debated whether aggregation of TDP-43 in the cytoplasm of ALS and FTLD-U patients causes neurodegeneration due to formation of toxic protein aggregates (gain of function) or to the translocation of TDP-43 from the nucleus, which represents its physiological location, to the cytoplasm (loss of function), or both. Our results show that delivery of exogenous TDP-43 into the cytoplasm occurs in the absence of a significant loss of endogenous TDP-43 in the nucleus. Indeed, the images acquired after delivery show cytoplasmic TDP-43 in the absence of detectable clearance of nuclear TDP-43. Thus, the non-amyloid, amorphous aggregates formed from TDP-43 are inherently highly toxic to neuronal cells, indicating that a gain of function mechanism caused by TDP-43 deposits is effective in such pathology. The data do not exclude that a loss of function mechanism originating from the cellular mistrafficking of TDP-43 also contributes to pathology, but shows the inherent toxicity of TDP-43 aggregates.

In conclusion we have shown, using bacterial IBs containing aggregated TDP-43 as a model system, that both FL and Ct TDP-43 aggregates consist of non-amyloid assemblies that have an intrinsically high ability to cause neuronal dysfunction when delivered into the cytoplasm, contributing to elucidate the pathogenesis of TDP-43 proteinopathies such as FTLD-U and ALS.

## Supporting Information

Figure S1 **CR and ThT binding of wt AcPDro2 IBs and C43S AcPDro2 IBs.** (A) Absorbance spectra of wt AcPDro2 IBs+CR (solid line), CR (dashed line) and wt AcPDro2 IBs (dotted line). (B) Absorbance spectra of C43S AcPDro2 IBs+CR (solid line), CR (dashed line) and C43S AcPDro2 IBs (dotted line). (C) Difference absorbance spectra obtained for wt AcPDro2 IBs (blue) and C43S AcPDro2 IBs (red). (D) ThT fluorescence spectra in the presence of wt AcPDro2 IBs (blue) and C43S AcPDro2 IBs (red). The CR and ThT analyses show that IBs containing the destabilised and amyloidogenic C43S mutant of AcPDro2 bind CR and ThT more markedly than IBs formed after expression of the less stable and less amyloidogenic wt AcPDro2. The C43S AcPDro2 IBs, therefore, can act as a positive control showing the presence of amyloid-like aggregates in IBs arising from the expressed protein.
(TIF)

Figure S2 **Amide I regions of FTIR spectra of wt AcPDro2 IBs (blue) and C43S AcPDro2 (red).** The analysis shows that IBs containing the destabilised and amyloidogenic C43S mutant of AcPDro2 have a large amount of β-sheet structure with respect to IBs formed after expression of the less stable and less amyloidogenic wt AcPDro2. The C43S AcPDro2 IBs, therefore, can act as a positive control showing the presence of amyloid-like aggregates in IBs arising from the expressed protein, confirming the CR and ThT analyses.
(TIF)

Methods S1 **Description of the methods involving preparation and analysis of wt AcPDro2 IBs and C43S AcPDro2 IBs.**
(DOC)
