# Supplementary figures and images for: TDP-43 Inclusion Bodies Formed in Bacteria Are Structurally Amorphous, Non-Amyloid and Inherently Toxic to Neuroblastoma Cells
Source: PLoS One. 2014 Jan 30;9(1):e86720. doi: 10.1371/journal.pone.0086720 (PMC3907574; doi:10.1371/journal.pone.0086720)

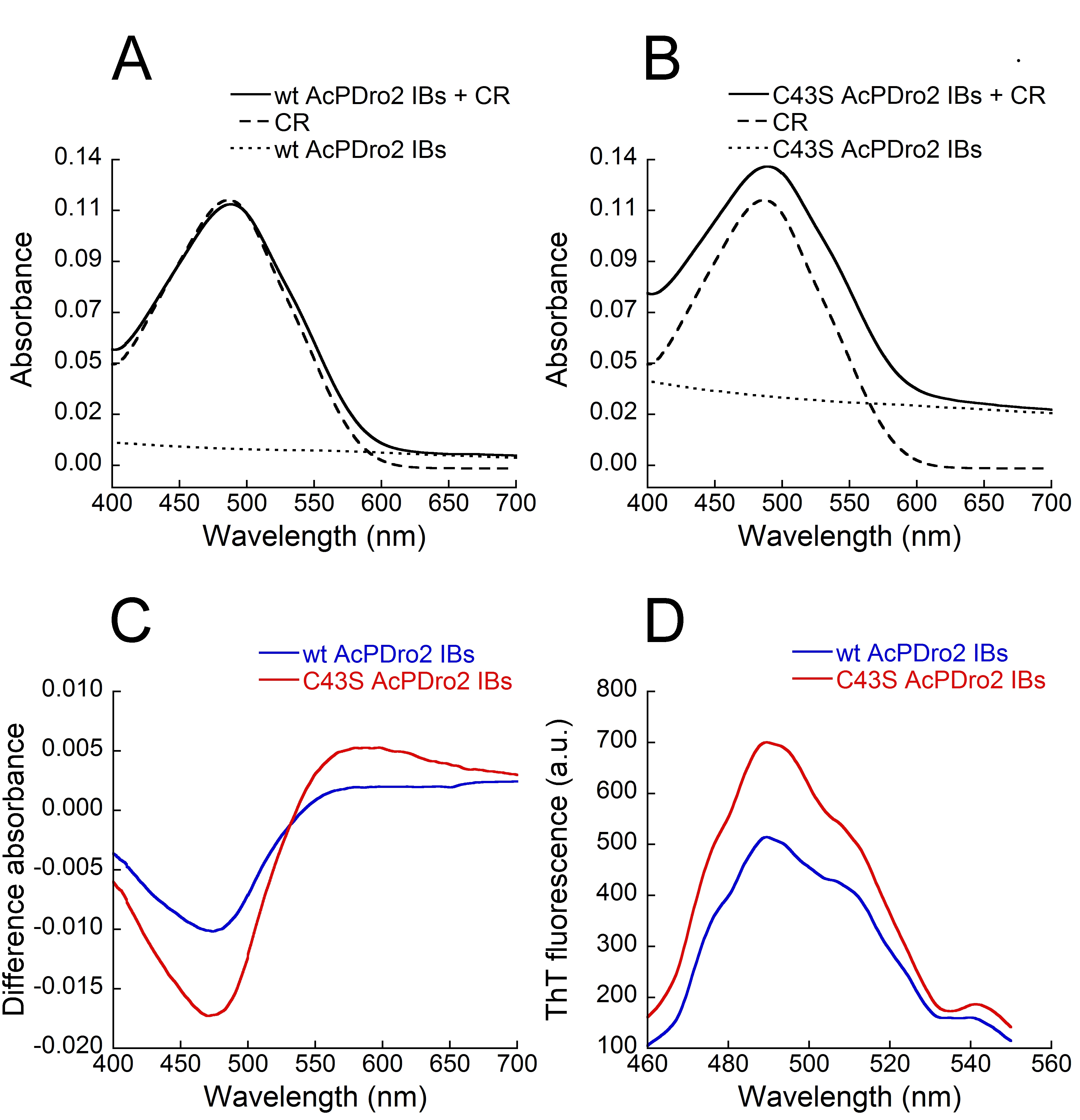

Supplement: Figure S1 — CR and ThT binding of wt AcPDro2 IBs and C43S AcPDro2 IBs. (A) Absorbance spectra of wt AcPDro2 IBs+CR (solid line), CR (dashed line) and wt AcPDro2 IBs (dotted line). (B) Absorbance spectra of C43S AcPDro2 IBs+CR (solid line), CR (dashed line) and C43S AcPDro2 IBs (dotted line). (C) Difference absorbance spectra obtained for wt AcPDro2 IBs (blue) and C43S AcPDro2 IBs (red). (D) ThT fluorescence spectra in the presence of wt AcPDro2 IBs (blue) and C43S AcPDro2 IBs (red). The CR and ThT analyses show that IBs containing the destabilised and amyloidogenic C43S mutant of AcPDro2 bind CR and ThT more markedly than IBs formed after expression of the less stable and less amyloidogenic wt AcPDro2. The C43S AcPDro2 IBs, therefore, can act as a positive control showing the presence of amyloid-like aggregates in IBs arising from the expressed protein. (TIF) [file pone.0086720.s001.tif]

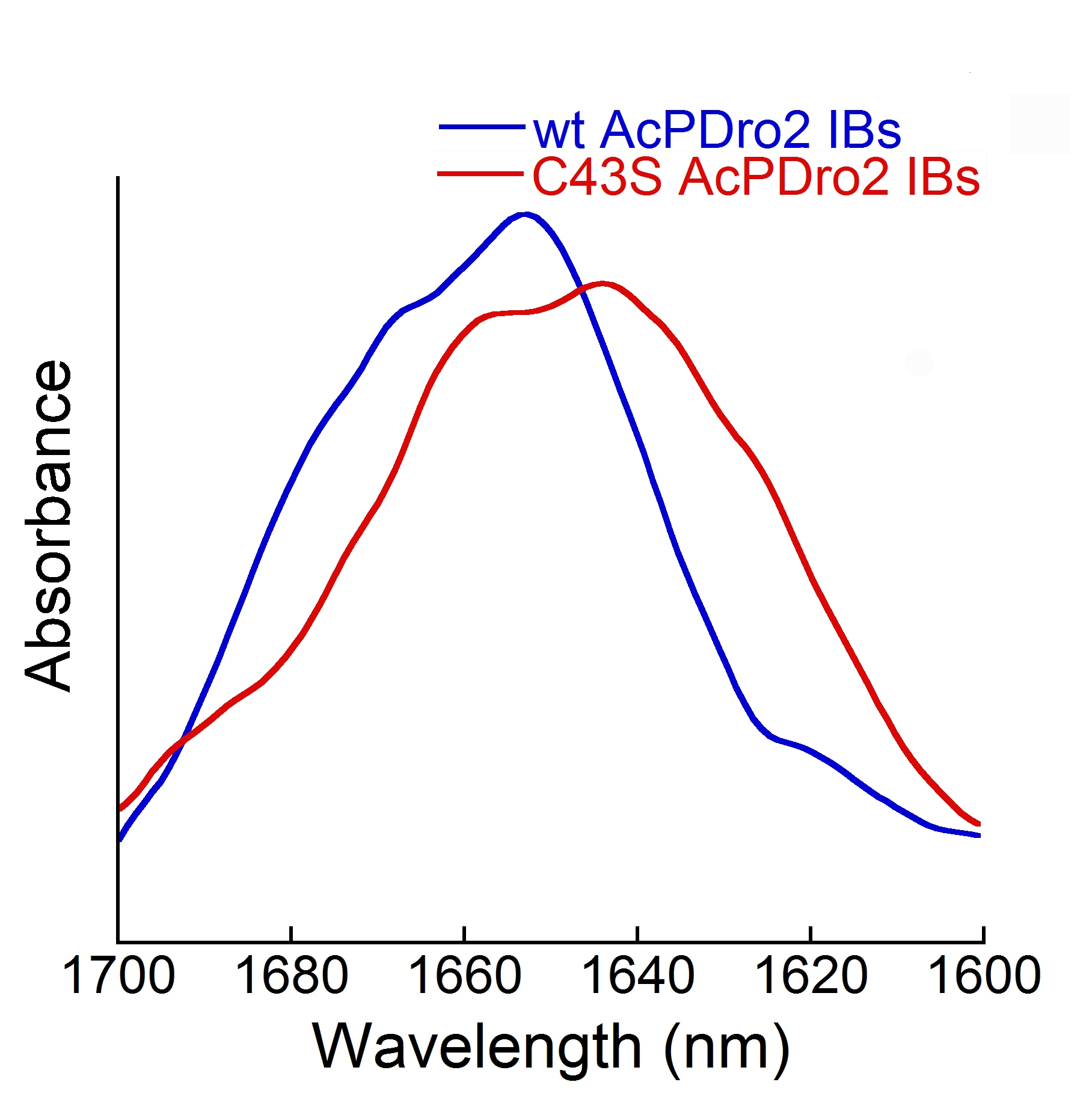

Supplement: Figure S2 — Amide I regions of FTIR spectra of wt AcPDro2 IBs (blue) and C43S AcPDro2 (red). The analysis shows that IBs containing the destabilised and amyloidogenic C43S mutant of AcPDro2 have a large amount of β-sheet structure with respect to IBs formed after expression of the less stable and less amyloidogenic wt AcPDro2. The C43S AcPDro2 IBs, therefore, can act as a positive control showing the presence of amyloid-like aggregates in IBs arising from the expressed protein, confirming the CR and ThT analyses. (TIF) [file pone.0086720.s002.tif]
