# Supplementary material for: TDP-43 Inclusion Bodies Formed in Bacteria Are Structurally Amorphous, Non-Amyloid and Inherently Toxic to Neuroblastoma Cells
Source: PLoS One. 2014 Jan 30;9(1):e86720. doi: 10.1371/journal.pone.0086720 (PMC3907574; doi:10.1371/journal.pone.0086720)
Supplement: Methods S1 — Description of the methods involving preparation and analysis of wt AcPDro2 IBs and C43S AcPDro2 IBs. (DOC) [file pone.0086720.s003.doc]

**Methods S1**

The mutation C43S was carried out in the gene encoding AcPDro2 using the QuickChange site-directed mutagenesis kit from Stratagene (La Jolla, CA, USA) according to the manufacturer’s guidelines, that were 95 °C for 30 s (1 cycle), 95 °C for 30 s, 55 °C for 1 min, 68 °C for 6 min (18 cycles). The correct insertion of the mutation was verified by DNA sequencing.

Cultures of *E. coli* BL21 cells transformed with the plasmids pGEX-4T/wt AcPDro2 and pGEX-4T/C43S AcPDro2 were grown overnight at 37 °C in LB medium with 100 μg/mL ampicillin under vigorous shaking. The cells were then diluted 1:10 in fresh medium and grown at 37 °C in LB until OD_600nm_ ~ 0.6. After protein expression induced for 2 h using 1 mM IPTG, cells were harvested by centrifugation and the IBs formed from cells expressing GST/wt AcPDro2 (wt AcPDro2 IBs) and GST/C43S AcPDro2 (C43S AcPDro2 IBs) were purified as described for TDP-43 IBs and control IBs in *Materials and Methods* of the main text.

Wt AcPDro2 IBs and C43S AcPDro2 IBs were prepared at the concentration of 1.0 mg/mL and their interaction with CR was assessed following the procedure and spectra analysis used for TDP-43 IBs and control IBs.

ThT fluorescence spectra of wt AcPDro2 IBs and C43S AcPDro2 IBs prepared at the concentration of 0.5 mg/mL were acquired similarly to that of TDP-43 IBs and control IBs.

Wt AcPDro2 IBs and C43S AcPDro2 IBs were prepared at a concentration of 40 mg/mL in D_2_O and the FTIR spectra were obtained as to that of TDP-43 IBs and control IBs.
